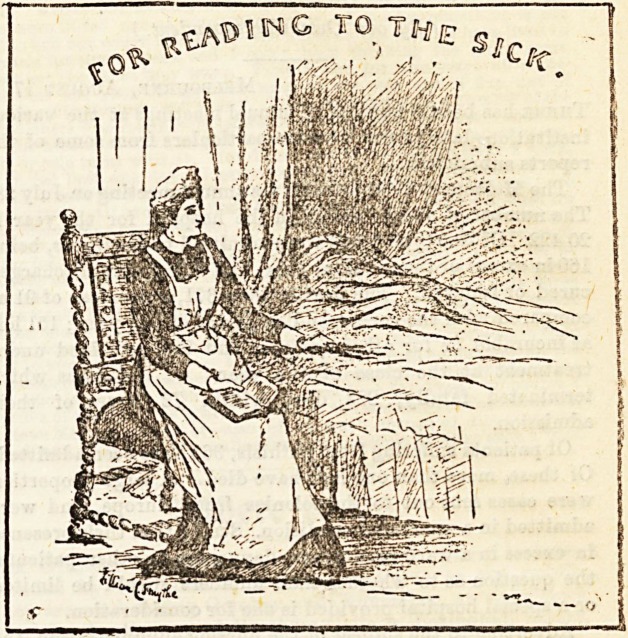# The Hospital Nursing Supplement

**Published:** 1891-10-03

**Authors:** 


### "The Hospital" Nursing Mirror.

Being the Extra Nursing Supplement of "The Hospital" Newspaper.

Contributions for this Supplement should be addressed to the Editor, The Hospital, 140, Strand, London, W.C., and should have the word "Nursing" plainly written in left-hand top corner of the envelope.

### En Passant.

**Lowestoft Nurses' Association.**—Sir Philip Cunliffe Owen took the chair at the third annual meeting of this Association, held on September 12th. During the year forty-four cases were nursed by the nurses—Cowles, Berry, and Crossley. Those benefited are the working classes, but the nurses are not exactly district nurses, as they take full charge of the patients allotted them. The funds are satisfactory.

**Halifax Union.**—In July the Halifax Board of Guardians appointed a sub-committee to consider the nursing of the sick poor in the Workhouse. The Committee started by visiting two model infirmaries—Birmingham and Liverpool. Having seen what ought to be, they recommend their Board to entirely dispense with pauper help in the infirmary wards, to reorganise and enlarge the nursing staff, and appoint a trained Matron, who shall be responsible to the Board alone. The report goes sensibly into the details of how the staff of nurses should be increased at small extra cost, and is in every way a useful and satisfactory pamphlet.

**Short Items.**—Miss Florence Nightingale is staying in Buckinghamshire with her brother-in-law.—Miss Ballin, editress of *Baby*, and writer on health subjects, was married last week.—Two women in France, who acted as midwives without holding a diploma, have been fined 100 francs each.—Last week's *Lady's Pictorial* contained a long account of Lady Waterlow and her home, where she delights to welcome the Barts. nurses.—Miss Field has been lecturing at Birmingham on Miss Marsden's work amongst the lepers. Miss Marsden is in need of funds.—Messrs. Simpson and Duckworth's design for the new Nurses' Home at Blackburn has been accepted. There will be thirty rooms for nurses, besides the Sisters and servants' rooms.—Miss Deane and Miss Lancashire have been lecturing for the Rural Branch of the Queen's Jubilee Institute.—Miss C. Lumsden lately held a party at Aberdeen Children's Hospital to celebrate the 14th anniversary of the opening of the institution.

**The Lay Press and the Nurses.**—"All is fair in love and war," and the press seems to think that all is fair that will fill their columns in the dull season; but a Glasgow contemporary has drawn wrath on itself for its persistent and ill-judged attacks on the Royal Infirmary. The absolute ignorance of the managers of the paper was shown when they proposed that the nurses should have the same food as the patients: beef tea, fowls, and, we suppose, gruel and port wine; all to be paid for by the ratepayers. The nurses certainly wouldn't like this, and why should healthy women be kept on dainty food at the expense of the public? If any of the nurses really wrote the letters which have been appearing in the *Mail*, we are shocked at the disloyal tone and carping spirit they have fostered in their midst; but the letters betray such ignorance of nursing matters that we are inclined to believe them the work of outsiders, in spite of the fact that they are all signed by some such title as "A Ward Nurse." Even after the managers have instituted an inquiry and are doing their best to learn the wishes of the nurses, the letters continue to appear in the paper. This shows a lack of courtesy all round, and is most unfair. We appeal to all loyal and lady-like nurses in Glasgow to try and secure redress without lowering themselves further in the eyes of their fellow-workers.

**Nurses for the Middle Classes.**—A good suggestion, but not a new one, was made in a letter lately addressed to the *Glasgow Herald*. The writer said: "Everyone knows the value of a trained nurse in illness, and those who have had to hire such know the great expense incurred thereby. Indeed, the expense is so great that many of us who feel we would be the better of some help in trouble have to go without on this account. Now, all know as well as I do, that we middle-class people are very proud, and will not accept of help in charity. But at the same time we middle-class people are very liberal in charity and donations. The rich can get a nurse, because they can afford to pay for one; and the poor, for they get one gratis. Now, I want to say that we have two valuable training homes for nurses, and I would like to suggest that perhaps some arrangement might be made with them that families who subscribe annually, say £1 or 10s. a-year, could in time of need have a nurse at 10s. a-week. This, along with doctor's bills and medicine, is heavy enough for people with moderate incomes." In America, where the nurses arrange and take their own fees, it is not unusual for a first-class nurse who has had a run of good cases to voluntarily undertake a case at two or three dollars a-week, when she knows the family cannot afford more.

**Male Nurses.**—We have always upheld male nurses, and felt aggrieved that doctors should as a rule regard them with distrust; but we are not so surprised since a male nurse has taken to writing in *Harper's* on medical mistakes. This is the style: "I helped another practitioner, in good and regular standing, to examine a man's heart. He found a pretty bad wheeze in the left side. I had to nurse that man. He had been on a bat, and all on earth that ailed him was that spree, but he got treated for heart trouble. It scared the man almost to death. I'd learned how a heart should sound, so one day I tried his. He was in bed then, and it sounded all right, so when the doctor came in I took him aside and told him that I didn't want to interfere, but that man was scared about to death over his heart, and it seemed to me it was all right—sounded like other hearts—and his pulse was all right too. The doctor was mad as a March hare. Well, to make a long story short, the fina discovery was—the man don't know it yet, and he is going around in dread of dropping off any minute with heart failure—that at the first examination the man had removed only his coat and vest, and his new suspender on his starched shirt had made the squeak. That is a cold fact, and that man paid over eighty dollars for the treatment he had for his heart." The male nurse seems utterly unconscious how he betrays not only his own ignorance but his own ill-feeling in these stories. There is another tale of a little Jap, who replied in the affirmative when he was asked if he ever spat blood: "Common sense told me the boy's lungs were all right; but it was none of my business, and so I watched him treated, off and on, for lung trouble for over a month before I got a chance to ask him any questions. Then I asked, incidentally, 'What made you spit that blood that time, Gihi?' 'I didn't know I ought to swallow him,' he replied, wide-eyed and anxious. 'Dentist pull tooth. He says to me, "Spit blood here." I do like he tell me. Your doctor says ver' bad for lungs, spit blood. Next time I swallow him.'" This is fortunately too good to be true.

### The Cyprus Society.

### Educational Work.

The extension and improvement of the hospital and educational work now carried on in the island of Cyprus are the main objects of this Society, at work here in London, and counting amongst its active members Englishmen who have been resident in the island, and, accordingly, know what reforms are needed, and the special kinds of help which would be well received by the Cypriots themselves. The Society's programme of hospital reform contemplates (1) the building of a cottage hospital in the district of Kyrenia; (2) developing a system of locomotive dispensaries and ambulances; (3) the training of native nurses for the hospitals which now exist, or may hereafter be established. In a former article these projects were examined in detail. We turn now to the educational efforts of the Society.

In this department, more, perhaps, than in any other, it is a condition of success that the local, race, and religious feelings of the inhabitants should be considered and respected. Too much credit cannot be given to the chief movers in this whole enterprise for the tact, sagacity, and charity they display on these points. In their case history has not been written in vain, and the world has not advanced without carrying themselves onward in its progress. The Society is to be commended for so strongly emphasising that it works for those of all races and of all religions in Cyprus, but is still more to be commended for the declaration, again and again repeated, that proselytism is not its aim or purpose or desire. About three-fourths of the inhabitants belong to the orthodox Greek Church; their Archbishop, when he comes to London, is cordially received by our own Archbishop, takes part in the services in our churches, and is recognised as the honoured representative of our fellow Christians of another great branch of the Christian family. To say that those under his spiritual jurisdiction have no need of conversion to the Christian faith seems indeed a bruiser, and yet, even in recent times, some well-meaning and narrow-minded people—in their proselytising labours—have strained almost to breaking the bonds of fraternal unity between ourselves and the Greek Church. We are pleased to find this society not only recognising the spiritual jurisdiction of the Archbishop of Cyprus, but securing his valuable sanction and co-operation in all they undertake. Bishop Blythe, our own Bishop for members of the English Church in the East, is also amongst those assisting in the work of the Society.

An Inspector of Schools is numbered amongst the officers which the Government has appointed for Cyprus. This post is admirably filled by the Rev. J. Spencer, who continues to get the best results from the present unsatisfactory school machinery, is well received by the officers of all existing schools, and has suggested the establishment of a school at Nicosia, the capital, for the higher education of boys, many of whom, he hopes, will become teachers in the elementary schools. There are at present elementary schools, aided by Government grants, in most of the villages of the island, but educational affairs, as will be shown directly, are at a low mark indeed, and real improvement is hopeless till the teachers themselves are better trained. Even a Cypriot priest, or "papa," is, as a rule, a peasant, educated only so far in advance of his flock as to enable him to read and write. Both education and religion must languish under these circumstances, and we are sorry to hear that many Bishops, themselves enlightened, and awake to the need of reform, almost despair of improvement. It will be a noble work for this Society to replace that despair by hope, and see that the hope is fulfilled.

The number of Christian elementary schools receiving Government aid is 241. (This does not include six Roman Catholic mission schools in Nicosia, Larnaca, and Limassol, which may be called private schools). But 34 of these schools were closed (temporarily) for want of funds to carry them on. Only 20 of the whole number are "girls' schools." The schools in operation employ 248 teachers—216 masters, 32 mistresses; the average annual salary being £29 10s. In these same schools the pupils number 9,473—7,886 boys, 1,076 girls. The average number of scholars for each school is 46, and the average daily attendance 38.

With the salary just mentioned (£29 10s.), educationalists at home would be prepared to hear of any eccentricity in the method of instruction. It is an eccentricity, no doubt, to have no particular method whatever, and when this is the rule rather than the exception, the surprising thing is that the work of education gets done at all. Two of the masters, it appears, have drawn attention to themselves, and made some commotion in the whole educational world of Cyprus by as tartling innovation, setting up and maintaining a definite rule and course of elementary education. These men may well be reformers amongst comrades who take up school work without any knowledge of their duties, and without any idea of method in the performance of them.

Forty pounds a year is the average cost of maintenance of an elementary and aided Christian school in Cyprus. Of the total cost involved by the 207 schools (*i.e.*, omitting fourteen, closed for want of funds), the Government grant provides 27 per cent., and voluntary contributions 73 per cent. There seems in every way a strong claim for larger state aid, the voluntary contributions are obviously doing their best, but the schools are at their worst. "Unless State aid can be increased," writes Mr. Spencer, "the schools will continue to be liable to frequent changes of masters and temporary closing, and cannot assume a permanent character. There is no hope of the schools becoming permanent institutions unless the masters are properly remunerated, and so are content to remain at their posts and take an interest in their work. And this will never be the case while the Government aid bears so small a proportion to the total cost of the schools as it does at present." There are besides, 35 additional Christian schools which receive no Government grant. By the inhabitants an effort is shown to give prominence to the important work of education, but the people are poor and behind the times, and sadly need the helping, guiding, strengthening hand of the philanthropist.

(*To be continued.*)

### Hints to Nurses.

By William J. Rugg, M.R.C.S.Eng., L.R.C.P.Lond.

It is most essential that nurses should have a knowledge of the various stools which are characteristic of certain morbid conditions, as it is not always practicable for a medical man to examine the fæces himself in every case, and the nurse is generally asked as to their appearance. Perhaps from neglect or ignorance she is unable to give a satisfactory answer, or from a sense of false shame replies that they are "All right." It may, therefore, not be out of place in these columns to draw attention to the colour and appearance of certain stools which may aid the doctor in his diagnosis. Perhaps the most important stool for the nurse to recognise is the "typhoid" stool; this is the colour of pea soup, pultaceous, very offensive, and which, on standing, divides into two layers, the upper layer consisting of fluid containing salts and albumin, whilst the lower layer consists of particles of undigested food, epithelial cells, and shreddy mucous. "Cholera" stools are likened to rice-water, *i.e.*, watery, colourless, nearly odourless, and containing white flakes, which are seen floating in it. "Dysenteric" stools can hardly be mistaken, owing to the blood they contain, and which are very scanty and shiny, with a most offensive odour.

Whitish-clay colour or "biliary" stools are hard and very offensive, owing to the absence of bile, which acts as an antiseptic to the contents of the intestine. When there is hæmorrhage into the stomach, or high up in the intestines, we get "farry" stools, which are due to the admixture of the blood with the fæces; while, if the hæmorrhage be lower down in the bowel we get the characteristic, "Black-currant jelly" stool. Again, when there is some catarrh of the intestine, we get the "mucous" stool; while "fatty" stools in pancreatic disease, and "lienteric" stools, containing scarcely altered food, are all of aid to the doctor in forming a correct diagnosis to his case.

It should not be forgotten by the nurse that the stool may contain "gall-stones" or "thread worms"; or that, if the patient is taking certain medicines, they may impart a characteristic colour, as, for instance, the "black" stool in a person taking iron or bismuth, or the "green" stool of one taking calomel.

Lastly, the shape of the stool may be of service in bringing to light a morbid condition as the "tape-like" or "pipe-stem-like" stools which occur when there is stricture or new growth laid down in the bowel, and which shape is due to mechanical influences.

### Another Letter on India.

Dear Mr. Editor,—I have not last week's Hospital by me now, but as far as I remember a (would-be Indian) nurse desired some information on India, and as I have come from, and have done six months nursing in a large hospital there, I may be able to enlighten your questioner a little.

Your "special correspondent" in this week's issue deals only with the Nursing Fund to which she belongs, viz., Lady Roberts', but there are plenty of other Institutions which are only too glad to have a well-trained English nurse, and most pay the passage out, but bind you to three years' agreement, and on completion, pay the return passage.

There are in Bombay alone three hospitals, the European General, which is a Government one, and the nursing is under the All Saints' Sisters. They have, I know for a fact, trained nurses from home, for nursing in the wards, and private work, and they also train probationers. The only objection is the mixture of Europeans and Eurasians, but I expect in most hospitals, even in England, there is a mixture of some sort.

The Jamseeji Hospital is for natives, and the nursing is also managed by the All Saints' Sisters. They have European nurses, and were talking of training native girls as probationers, so I heard.

The Cama Hospital is another under European management, and is for Parsees only.

In Poona, not seven hours' journey from Bombay, there is the David Sassoon Hospital, for Europeans and natives (seperate wards), the nursing worked by the Sisters of the Wantage Society.

There are hospitals in Madras, Calcutta, Karachi, and in heaps of other places, quite apart from Lady Roberts or Lady Dufferin's Funds. The former is only for military nursing, and the latter for natives, and I fancy only for women.

The Lady Roberts' Nurses are much better off in more ways than one, than their fellow Indian Nurses. The salary is nearly six times more than the general run, they are always in a Hill Station I believe, have shorter hours of duty, ward servants, and last but not least, all are gentlewomen, and are treated as such.

At the hospital in which I was nursing, the hours were arranged as follows: Chota Hazri, at 6.30; go on duty at 7 a.m.; breakfast at 10; slight lunch at 2; tea at 5; and dinner at 8 p.m., when day and night nurses all dined together, and the day nurses came off duty and night nurses went on at 8.30 till 7 a.m. Of fever cases I have nursed plenty, some slight, and others very tedious and long, but the temperature of the worst did not exceed 106° as a rule—one exceptional case, of "enteric" proving fatal, being 110° before death. I have had, in one case, three ice packs in one night, nourishment every ten minutes, temperatures every two hours, and have twice been on duty twenty-four hours at a stretch, but have had the satisfaction of seeing my patient recover. Ordinarily our time "off duty" daily consisted of one hour and a-half, and once a week, a "long pass," from 2 p.m. till 6.30 p.m., when we came on duty till dinner time, and half a day off every alternate Sunday.

The heat of Bombay is well known all over India (though it is not so bad as in some places I've been in), and in the summer nights we used to lie gasping under our mosquito nets, listening to the "singing" of the mosquitoes, and envy our more fortunate sisters in the hills; and, *D.V.*, if I am lucky enough, and have interest enough, I hope to be one of those same said fortunate ones—when I have attained the prescribed age. I am home from India, especially to have a thorough training in nursing, and though I love the Mother Country, I am looking forward to the day when I once more sail for the gorgeous (save the mark!) East. We grumble at the heat, when we are there, and we complain bitterly of the cold when we are here, but 'tis human nature, and that is excuse enough. If your last week's enquirer would care to know any more particulars than I have already mentioned, I shall be most happy to supply her with any information, if she will write to me personally.—Yours truly,

Old Indian.

### THE HUSBANDMAN.

The heart of man is like a field, which requires careful and constant cultivation. Unfortunately, the resemblance is very great in the particular that weeds spring up if we only leave them alone for a very short time. Grain and fruit will not grow on a poor, neglected soil; yet, be it never so stony and dry, there will soon be a plentiful crop of thorns and thistles. A good husbandman then sets to work with a will; he rises early, and late takes rest, and eats the bread of carefulness. He breaks up his ground with plough and harrow, he sows good seed, hoping it will bring forth fruit, some twenty, some sixty, some an hundredfold.

He knows, however, that it will be but lost labour unless the Lord works with him, sending the rain and causing the sun to shine and ripen the fruits or the swelling grain.

Are we good husbandmen? It is well, if from our youth we have tended and kept our hearts in order; if not, let us look at once to it aand do our part, that we may have a bountiful harvest when we can no longer work. We may, perchance, have been idle and careless, and let the weeds of foolishness, or obstinacy, or deceit, choke our hearts and wither up the love to God and our neighbour, which ought to be flourishing there." Our Heavenly Father sees and grieves over our barrenness, and would have our wilderness to blossom like a rose. He therefore sends us crosses and disappointments in life as gentle hints to lure us back to our duty. Should these fail, He breaks up our fallow ground Himself. By sickness, by poverty, by trouble, He prepares our hearts to receive the showers of blessings which he has always ready for those who love Him. Let us take this husbandry in a right spirit, and be thankful for the training which fits us to do our part on earth profitably. We shall pass our days cheerfully here, working for and with Him who is the great husbandman, and this life ended, rejoice in the fair fields of Paradise, where stands the Tree of Life, whose leaves are for the healing of the nations.

"As the hardy oak is growing,
Howsoe'er the wind may blow;
As the tired stream is flowing,
Whether shines the sun or no;
Thus tho' storm winds rage around us
Should the strong plant, Duty, grow—
Thus with beauty or without it,
Should the stream of being flow."

### Notes from Australia.

*(By our Own Correspondent.)*

Melbourne, August 17.

There has been a number of annual meetings at the various institutions lately, and here are particulars from some of the reports submitted.

The Melbourne Hospital held its annual meeting on July 28. The number of cases treated in the hospital for the year is 20,422. Of this total 5,383 were admitted to the wards, being 160 in excess of the previous year, and 3,264 were discharged cured or relieved. The deaths were 671, a decrease of 91 as compared with the corresponding period of last year; 151 left as incurable or for other reasons, and 297 remained under treatment at the close of the year. Of the cases which terminated fatally, 228 died within 72 hours of their admission.

Of patients suffering from phthisis, 368 have been admitted. Of these, more than one-half have died. A large proportion were cases sent out to the colonies from Europe, and were admitted in a moribund condition. Seeing that their presence in excess in a ward may prove dangerous to other patients, the question as to whether their numbers should be limited or a special hospital provided is one for consideration.

Adverting to the subject of the nursing administration, the Committee have pleasure in stating that the training school for nurses is in full operation. Lectures to the staff are now in course of delivery by Dr. William Moore and the Lady Superintendent.

The Homœopathic Hospital held its annual meeting on July 27th, when a good balance-sheet and report were presented. The Chairman, in moving their adoption, said that the amount received for the services of nurses trained in the hospital was steadily increasing. Amongst the Government officials there was some difference of opinion as to the wisdom of maintaining a larger staff of nurses than were absolutely required in the hospital, but it was a point on which there was no difference of opinion amongst members of the Board. They were at one as to the wisdom of keeping up a large staff of lady nurses, as it was considered important by doctors practising homœopathy that they should be able to secure the services of nurses who had a practical training in homœopathic principles. He wished to speak also of the great value to the hospital of the Ladies' Aid Association, an idea brought from America by Dr. Ray, and which in its working had proved entirely successful.

The Eye and Ear Hospital reports 3,653 new cases treated during the year; total expenditure £2,768.

According to the report of the Austin Hospital for Incurables, during the year there have been 88 admissions viz., 57 men and 31 women, of whom 24 were suffering from cancer, and 34 from phthisis. The deaths for the same period numbered 58, viz., 40 men and 18 women, 18 dying from cancer, and 24 from consumption. In addition, 26 patients left to go to their friends; one was transferred to the Lunatic Asylum, and one was discharged for misconduct. On June 30th there were 90 patients in the hospital, viz., 55 men and 35 women. The total receipts for the year amount to £6,009; the expenditure to £5,946, of which £2,000 was spent on building the new consumptive wing.

At the Women's Hospital the annual meeting was held last week. The report set forth that the amount of work done in both departments during the past financial year had been both very extensive and satisfactory. The total number of patients under treatment during the year had been 2,828; of these, 1,270 were in-patients, and 68 cases had the fee of £1 paid for nursing them at their own homes. The mortality had been very low, the death-rate of the midwifery patients being only 0·58—about the lowest on record in the history of the hospital. During the year 48 medical students and 29 pupil nurses had attended the practice of the hospital.

The Melbourne District Nursing Society has had some new members elected on to the Committee, and they are infusing new life into the work. A "Cake Fair" is to be held this week in aid of its funds. Cake fairs are very successful in Victoria, and much nicer than bazaars. Not only are all sorts of cakes on sale, but butter, eggs, poultry, fruit, and light provisions generally. It would surely be a good plan to introduce cake fairs to the notice of London ladies during the coming season.

The Charities Commission has had two minor excitements lately; in one case contradictory evidence as to whether the Medical Officer of the Castlemaine Hospital and the Committee were competent or not, and the other with reference to the death of a patient at Horsham Hospital, who, owing to the fact that there is no resident officer, died before he received medical attendance.

Great interest has been displayed in the trial of Colston for the murder of Mr. and Mrs. Davis. The jury have returned a verdict of "guilty," in spite of the fact that a very clever plea of insanity (general paralysis of the insane) was brought forward, and supported by this style of evidence. Dr. Mullen: Do you understand the significance of the eye symptoms? Dr. Youl: No, I do not. Do you understand the facial symptoms?—Yes. Which is the motor nerve of the face?—The fifth. Do you mean to swear that the fifth nerve is the motor nerve?—Yes. Well, any student would be "plucked" for such an answer. What does the seventh nerve do?—I don't pretend to a minute knowledge of anatomy. It is impossible for me to have such a knowledge. Mr. Finlayson: Is this a university lecture, or are we all going back to school again? Dr. Mullen: This is very important. The question of general paralysis has never been thoroughly raised before. (To the witness)—Do you know in what part of the brain general paralysis first begins? No.—Do you mean to tell me that with this want of knowledge you are competent to give an opinion on a doubtful case? Yes. I have a wide practical experience of insanity. I have examined large numbers of brains, and those of insane people showed no peculiar symptom.—Did you examine them under the microscope? No.—Have you ever seen the symptoms observable on Colston on any person who is not a general paralytic? Yes.—What became of that person?—I know that he is not a general paralytic. To Mr. Finlayson.—I have forgotten the names and functions of the different nerves, though when I was about the age of Dr. Mullen I dare say I knew all about them.

### Presentations.

The medical and nursing staff at St. George-in-the-East Infirmary spent a very pleasant evening on Saturday, the 12th inst., when the Matron, on behalf of the officials and nurses, presented the Medical Superintendent, Dr. Harris, with a handsome pair of silver fish-carvers and a set of silver salt-cellars on the occasion of his leaving the infirmary for East Dulwich. The proceedings were enlivened by vocal and instrumental music, and the room and tables were most tastefully decorated by the nurses. St. George has to thank Dr. Harris for many improvements, and his resignation is deeply regretted by all concerned. The entire nursing staff is now fully trained.

Miss Rachel Lumsden, the Hon. Superintendent at the Royal Infirmary, on September 14th, presented the six senior nurses of the Aberdeen Children's Hospital with a handsome brooch each, to commemorate the fourteenth anniversary of the opening of the Hospital, and to mark her appreciation of the nursing in the Institution where she was formerly Matron.

### Everybody's Opinion.

[*Correspondence on all subjects is invited, but we cannot in any way be responsible for the opinions expressed by our correspondents. No communications can be entertained if the name and address of the correspondent is not given, or unless one side of the paper only be written on.*]

### RELIGION AND DISEASE.

The Rev. T. G. Headley writes: We are often told that religion is beautiful and priceless, but theological dogmas that have outlived their natural term of existence are often as destructive to the intellect and to the soul as material putrefactions to the bodily organism. But when these two radical and opposite statements are both fully admitted, yet nevertheless they need very careful treatment, lest, to use another simile, in pulling up the tares we pull up the wheat also. In answer to this it may be said there needs no pulling up at all, and that it is only necessary to avoid theological dogmas that have outlived their natural term of existence. But the very fact of its being admitted that they have outlived their natural term of existence is an admission that they are not dead but are actually living and destructive to the health of the intellect and of the soul in like manner as material putrefactions are destructive to the bodily organism. So that when this is admitted, it would naturally be supposed that in like manner, as it is deemed absolutely necessary for the health of the bodily organism that material putrefactions should be treated for their removal, and not ignored to work their own deadly ill, so likewise it would be deemed absolutely necessary for the health of the mental and spiritual organism that what is destructive to their health should likewise be treated for its removal and not left to work its own deadly way. And yet, marvellous to relate, there are men who are keenly alive to the ills of our bodily organism and the folly of neglecting them, who nevertheless are oftentimes absolutely hostile to any attempt being made to remove theological dogmas that literally poison the very source of morality and religion at its fountain head.

### NURSES AND STUDENTS.

Sister Mona writes: It is easy for us to laugh at the youthful conceit of the student of Guy's who thinks his behaviour to the nurses is of the foremost importance in the nurses' lives, and that he is capable of directing their studies. Most of us see month after month fresh batches of raw students coming in to dress their one or two cases, and generally quite ready to learn their work from the nurses. We pass our three years of perpetual work in the wards; they their three months of most limited attendance. In one well-known northern hospital the students are only allowed in the wards for two hours a day. In another a dresser has two and a half beds allotted to him! Now, how can these students get the practical knowledge they need? I remember Sir Andrew Clark stating before the Lords' Committee that he came up from Edinburgh with his degree, but with all the practical knowledge to be gained. This I can imagine, for an Edinburgh student who goes up for his final next July told me a month ago, when I spoke to him of massage, that he heard of that treatment for the first time. Instead of laughing at the students, I think we ought to back them up in every way when they try to gain the experience we are so blessed in having.

### THE LONDON HOSPITAL.

"Sister" writes: Permit me to correct two mistakes in your article, "To Would-be Nurses." Probationers here received £12 the first year and £20 the second year; not £10 and £15 as stated. The certificate is given to everyone who serves the two years satisfactorily, whether they pass the examination or not.

### NURSES' FOOD.

"L." writes: On looking over my Hospital for last Saturday, I see some more ill-fed nurses are complaining. I wonder something is not done to find out which is in fault—the nurse or the diet. I have lived in hospitals for eleven years, and I can't help thinking the more some nurses get the more they want. First I was for two years at a hospital that the pros of my time used to say was one of five, out of London, with the crrrect "tone." There were certainly a number of the probationers who really were ladies, so much so that they never asserted it. For breakfast there we got tea and bread and butter; never bacon, eggs, or cold meat with it. For dinner course mutton six days a week, and boiled beef one day. I never tasted roast beef all the time I was there. I have been Matron for the last year of a little hospital, and have three nurses. Of course a little hospital must cost more per head for diet than a large one need do. Of course, too, I am on my honour not to be extravagant. But I am certain that the nurses here are much better fed than the probationers were in the first hospital I was in, or than the nurses in two or three others where I have been charge-nurse of wards. I allow them for breakfast bacon four days, and eggs three days a week: half a-pound of cooked meat, and always pudding or tart for dinner, nicely cooked. And yet, with it all I have heard more grumbling about their rights and what they ought to have in this little place than I did in two years at B——. I must say I have much admiration for the Aberdeen student, mentioned also in your issue of the 12th, or of anyone else who is content with plain living and high thinking. Of course I approve of plenty—it is pitiful to read of the starving scholars that Dr, Norman McLeod tells us of—also of as much change of diet as we can afford, and after that of not allowing vulgar people to make such a fuss.

### Where to Go.

The Gresham Lectures begin on the 6th. These lectures, founded by Sir Thomas Gresham, are free to the public. They are read during October and November, at six o'clock p.m., in the theatre of Gresham College, Basinghall Street, in the following order, and by the lecturers named: Physic, Dr. E. Symes Thompson, October 6th, 7th, 8th, and 9th; Divinity, Rev. Henry E. J. Bevan, M.A., October 13th, 14th, 15th, and 16th; Astronomy, Rev. E. Ledger, M.A., F.R.A.S., October 20th, 21st, 22nd, and 23rd; Rhetoric, Mr. J. E. Nixon, M.A., October 27th, 28th, 29th, and 30th. The Thursday Ballad Concerts commenced at the Victoria Hall, Waterloo Road, last week. German Reed's entertainment at St. George's Hall, Langham Place, re-opened on Monday with a clever play by Walter Browne, and a delightful musical sketch by Corney Grain. The two shilling seats are excellent if you arrive at the hall in good time. Mondays, Wednesdays, and Fridays at 8 p.m., and Tuesdays, Thursdays, and Saturdays at 3 p.m.

### Wants and Workers.

The Committee of the Malmesbury Nursing Institute intends holding a bazaar in November to raise funds for a home for their nurses. The Secretary, Mrs. Chubb, The Priory, Malmesbury, will be very grateful for any articles which workers can send.

*Ashton-under-Lyne District Nurse* acknowledges, with thanks, parcels from Nurse Summers and "H. M." She would be glad of more old linen.

### Notes and Queries.

### Queries.

(50) Can any of your readers tell me of a home where a respectable woman, aged sixty, willing to make herself useful and do needlework, could be received for a permanency?—*A. P.*

(51) Would a trained nurse, by taking the matronship of a large refuge, get any nearer being Matron of a hospital; or would it be against her?—*R.*

### Answers.

(49) The warden of the Nursing Guild of St. Veronica is the Rev. J. Dixon, St. Peter's Home, Mortimer Road, Kilburn. The warden of the Guild of St. Barnabas is the Rev. J. Russell, St. Albans, Holborn.—*A Sister of the G.S.V.*

*Miss A. M. H.*—Your parcel for No. 2 competition safely received.

*Nurse Blackmoor.*—Your friend had better apply at the offices of all the large shipping companies. The only two lines we know which have taken trained nurses as stewardesses are the Inman, and the Wilson Line, Hull. In answer to your second query, apply to The General Domestic Servants' Benevolent Institution, 32, Sackville Street, Piccadilly, London.

*Sister Dorothy.*—It cannot be done. No institution will take you for three months unless you pay. You can go to the Whitechapel Infirmary for a year, neither paying nor being paid.

*Janet.*—Not in the least; simply because you were in such a hurry to get more jam to your bread you have dragged a noble institution in the mud. If you benefit, the patients will suffer. It is not the complaint, but the way in which it was made which angers us. Don't you know George Eliot's maxim, "Do not make your gain out of another's loss"?

*Nurse S.*—There seems to be an opening in Madeira. We have constantly had letters from nurses there. We do not advise Gibraltar. So much depends on introductions, and whether you hold a diploma for midwifery. Mrs. Mary Lamster, Seamen's Hospital, Funchal, Madeira, might possibly give you some information if you wrote to her.

*One of the First Thousand* and *A Mother of Ten.*—Next week.

### Kitty's Find.

### STRAYED AWAY.

She was Kitty at home, but when she put on her uniform of cap and apron, veil and cloak, she was Nurse Katherine. Somehow, with the more dignified name she also assumed another nature, sedate, thoughtful, and alert. Everybody knew they could trust Nurse Katherine; the most trivial details of the doctors' directions were absorbed, digested, and unerringly acted upon by this skilful nurse. Indeed, there were people who enviously pronounced her to be a mere machine who could not so much as think for herself.

"So much the better," said others who were wiser; "that's exactly what she should be so long as she's a nurse. When she comes to be a doctor-woman—which will be never—then she may begin to think for herself; meantime, let her remain as she is."

But the Kitty who packed her handbag, put herself into plain attire and sailor hat, and went off for a holiday, a real good time, was a heedless, careless girl brimming over with delightful anticipations.

"Nobody shall know where I am going, not even the home-people, for I shan't go there this time. I want an utter, upside down change."

And where did Kitty go?

Well, she took a ticket for nowhere in particular, and then she got out at the first place that struck her fancy, a sleepy, unknown, roadside station, where the porter, dozing in the sunshine, rubbed his eyes open as the train creaked in. In three minutes more the clatter of the departing train was dying away; the disturbed porter was lounging off to his sunny seat for another siesta, and Kitty, pushing back the sailor hat, drew a long breath as she stared round at

"The landscape winking through the heat."

Already she felt a thousand miles away from her hospital world, from the sorrow and sighing of town-life. It was an odd sensation being set down in an utterly unknown district; at a station where not a welcome greeted her; not a voice questioned her destination. It was freedom with a vengeance; but Kitty was undaunted, and sauntered leisurely—it would have been out of place in that lazy spot to be anything but leisurely—out of the modest station to find herself on the high road. A few hundred yards off she saw a hamlet of about a dozen cottages; but what was that faint aroma on the sunny air? Kitty's little nose sniffed intelligently.

"Hops!" she exclaimed jubilantly. "Well, that is lovely! I must be in the hop-country." And so she was, and that same trained nose conducted her unerringly until, suddenly, she walked right into a vast hop garden. It was a pretty sight, that scene under an intensely blue sky; the graceful bines; the picturesque-in the distance—hop-pickers; the heavily-laden wains lumbering off the ground to the oast-house.

"Come to pick, Miss, or to preach?" It was the bin-man who spoke.

"To pick!" was the laconic answer, on the spur of the moment.

"Well, we're remarkable short of hands, or leastways, I should say its a remarkable yield;" and Kitty was engaged on the spot, before she realised what she was doing. What a wondrous experience! thought the new hopper as she set to work with a will. The bin-man whistled as he watched her curiously.

At last Kitty slowed; she had grown tired. For the first time she glanced at her neighbours. They were a motley crew looked at closely. City faces were already tanned brown by the glorious summer sun, but they were strangely gaunt with deep hollows—starvation's work. Mingled among them were the children, giddy with the joy of being in the open. What a heaven the hop garden was to them; and Kitty smiled sympathetically as she watched the little ones suddenly leave off picking and rush for the bearers of the gallons of milk which the farmer's "good lady," a sweet motherly soul, had sent out to them. To those East-end mites the hop gardens were of a truth a Promised Land. They were so well treated as a rule by the hop growers that the small weazened faces relaxed their lines of sharp cunning in the sense of satisfaction that stole over their owners. But the women were pitiful enough to behold in their rags and their despair, as with an almost wolfish eagerness they picked away in order to earn the few shillings to take back with them to Whitechapel.

"Poor souls!" muttered Kitty, her tired arms falling limply by her sides as she regarded them compassionately; "can they ever have been young, and simple, and light-hearted? Were they ever girls?—Allie!" Her voice rose sharp and shrill, as suddenly there confronted her one of the very women her heart was aching over: a tall, languid figure; a grim, troubled face; eyes with a shamed droop of their lids, and matted hair, once fair, now dull ashen in hue from neglect.

"Allie!" repeated Kitty hoarsely.

"Oh, Miss Kitty! You won't tell!" there was a wail, and then the wretched woman, throwing her tattered apron over her head, sobbed aloud.

*(To be continued.)*

### Appointments.

[It is requested that successful candidates will send a copy of their applications and testimonials, with date of election, to The Editor, The Lodge, Porchester Square, W.]

St. George's Infirmary.—Nurse Penny has recently been appointed Assistant-Matron at St. George-in-the-East Infirmary. She was trained at the London, and for some years has been charge nurse at St. George's. She has the best wishes of all who know her for her success in her new post.

St. Kilda, Victoria.—Miss Davey has been appointed Matron of this Trained Nurses' Home in Australia. Miss Davey trained at University College Hospital, and emigrated to New South Wales to become Matron of the West Maitland Hospital, a post she has lately resigned.

### Amusements and Relaxation.

### SPECIAL NOTICE TO CORRESPONDENTS.

**Fourth Quarterly Word Competition commences October 3rd, 1891.**

The word for dissection for this, the FIRST week of the quarter, being

"WELCOME."

| Names. | Sept. 24th. | Totals. | Names. | Sept. 24th. | Totals. |
|---|---|---|---|---|---|
| Paignton | — | — | Christie | — | 44 |
| Psyche | 26 | — | Dulcamara | 26 | 475 |
| Hope | — | 47 | Nurse J. S. | 24 | 473 |
| Lightowlers | 24 | 512 | Qu'appelle | 26 | 514 |
| Wizard | — | 179 | E. M. S. | — | 68 |
| Wyameris | — | 46 | Jenny Wren | 23 | 467 |
| Dove | — | 46 | Carpe-diem | — | 65 |
| Punch | — | 181 | Grannie | — | 36 |
| Ivanhoe | — | — | Nurse G. P. | 16 | 29[illegible] |
| Tinie | — | 93 | Goodnight | — | 122 |
| Agamemnon | 25 | 495 | Gamp | — | 100 |
| Nurse Ellen | — | 86 | Charity | — | 104 |

### Notice to Correspondents.

**Results of Third Quarterly Word Competition will be given next week.**

All letters referring to this page which do not arrive at 140, Strand, London, W.C., by the *first post on Thursdays*, and are not addressed PRIZE EDITOR, will in future be disqualified and disregarded.

N.B.—Each paper must be signed by the author with his or her real name and address. A *nom de plume* may be added if the writer does not desire to be referred to by us by his real name. In the case of all prize-winners, however, the real name and address will be published.

## Figures and Tables

**Figure f1:**